# Crosstalk between autophagy and CSCs: molecular mechanisms and translational implications

**DOI:** 10.1038/s41419-023-05929-3

**Published:** 2023-07-08

**Authors:** Dai Li, Xueqiang Peng, Guangpeng He, Jiaxing Liu, Xian Li, Weikai Lin, Jianjun Fang, Xinyu Li, Shuo Yang, Liang Yang, Hangyu Li

**Affiliations:** 1 Department of General Surgery, The Fourth Affiliated Hospital, China Medical University, Shenyang, 110032 China; 2 Shenyang Clinical Medical Research Center for Diagnosis, Treatment and Health Management of Early Digestive Cancer, Shenyang, 110032 China

**Keywords:** Cancer screening, Cancer screening

## Abstract

Cancer stem cells(CSCs) play a key role in regulating tumorigenesis, progression, as well as recurrence, and possess typical metabolic characteristics. Autophagy is a catabolic process that can aid cells to survive under stressful conditions such as nutrient deficiency and hypoxia. Although the role of autophagy in cancer cells has been extensively studied, CSCs possess unique stemness, and their potential relationship with autophagy has not been fully analyzed. This study summarizes the possible role of autophagy in the renewal, proliferation, differentiation, survival, metastasis, invasion, and treatment resistance of CSCs. It has been found that autophagy can contribute to the maintenance of CSC stemness, facilitate the tumor cells adapt to changes in the microenvironment, and promote tumor survival, whereas in some other cases autophagy acts as an important process involved in the deprivation of CSC stemness thus leading to tumor death. Mitophagy, which has emerged as another popular research area in recent years, has a great scope when explored together with stem cells. In this study, we have aimed to elaborate on the mechanism of action of autophagy in regulating the functions of CSCs to provide deeper insights for future cancer treatment.

## Facts

- Regulation of autophagy can alter the stemness of CSCs to affect their survival.
- Mitophagy has its own unique metabolic reprogramming phenomenon.
- Immune combination therapy targeting autophagy can significantly enhance anti-tumor effects.

## Open questions

- Compared with differentiated cancer cells, what is the special role of autophagy in undifferentiated cancer stem cells?
- How mitophagy regulates the survival of cancer stem cell?
- What are the current difficulties in clinical transformation in the field of autophagy and stem cells?

## Introduction

Cancer stem cell are a small subset of cancer cells with stem cell properties, are highly proliferative and self-renewing, as well as possess multidirectional differentiation potential. This fraction of cells, although a small percentage, plays a key role in regulating tumorigenesis, progression, invasion, metastasis, resistance to radiotherapy, and recurrence. The CSCs hypothesis suggests that there may be a small fraction of cells with specific stem cell-like functions present in all cancers [1]. Most identified CSCs possess specific cell surface markers that are similar to those of the corresponding normal tissue stem cells. Therefore, non-specific surface markers can complicate the identification of CSCs. However, it is worth noting that the same CSCs may display different surface marker molecules. The same surface marker may be present on the multiple cancer cells; for example, CD133^+^ is not only a specific marker for AML, but also for brain [2] and liver CSCs [3]. The complexity of the different surface markers make therapeutic efforts difficult in the field of oncology, which has led to identification for more representative and specific surface markers. CSCs also have unique metabolic features that support its associated energy requirements and maintain their self-renewal, tumorigenic, and differentiation potential [4]. For instance, previous studies have suggested that CSCs are slow to metabolize and rely primarily on glycolysis to provide energy. In contrast to the differentiated cancer cells, CSCs maintain homeostasis by predominantly relying on the process of glycolysis to reduce the level of reactive oxygen species (ROS) [5]. However, studies have found that some CSCs are more inclined towards oxidative phosphorylation than glycolysis. For example, glioblastoma stem cells (GSCs) have a higher oxidative capacity and ATP levels than the differentiated glioma cells [6]. Breast CSCs (BCSCs) exhibit decreased lactate production and increased ATP levels. In addition, inhibition of mitochondrial biogenesis can result in reduced oxidative metabolism in BCSCs [7]. Therefore, the tumor microenvironment plays a key role in determining the metabolic phenotype of CSCs [8].

Autophagy is a catabolic process which is required for survival and function of organisms. The substances in the cytoplasm are able to enter directly into the lysosome for degradation. It can maintain intracellular homeostasis by eliminating the various dysfunctional organelles or damaged macromolecules. So far, three major classes of autophagy have been identified: macroautophagy, microautophagy, and chaperone-mediated autophagy (CMA) [9]. Autophagosomes undergo three main stages in the process of fusion with the lysosomes-initiation, extension, and maturation [10]. A number of reviews have already provided detailed insights into the various autophagy-related signaling pathways and hence these will not be presented here [11]. Unlike macroautophagy, which has traditionally been well studied, in recent years, several studies have been biased towards microautophagy and CMA [12]. Microautophagy essentially engulfs substances in the cytoplasm by isolating them, which in turn can degrade the lysosomes [13]. CMA degrades the damaged proteins to participate in homeostatic regulation under the stressful conditions [14]. Thus, the selective degradation of the lysosomal components by CMA reveals that it is also a selective autophagy [12]. Selective autophagy allows the cells to control the number of organelles in vivo once required. It possesses the ability to eliminate the dysfunctional pathogens by binding to the ubiquitin-proteasome system (UPS) [15]. However, in some cases, the overexpression or deregulation of autophagy can also lead to cell death [16]. Thus, autophagy plays two important roles. Autophagy has a distinct mission during different stages of cancer [17]. In the early stages of carcinogenesis, autophagy can reduce the emergence of the mutagenic factors and inhibit cancer development, whereas in the middle and late stages of carcinogenesis, autophagy can resist the stress conditions and inhibit apoptosis to maintain the survival of cancer cells [18].

Polypotency is an important property of CSCs, and autophagy can play a vital role in maintaining the polypotency of the stem cells [19]. Firstly, CSCs usually exhibited higher levels of autophagy, thus CSCs also often express elevated autophagic markers such as ATG5 and Beclin1 which reflected increased autophagic flux [20, 21]. In contrast, application of autophagy inhibitors have revealed a significant decrease in the number of CSCs along with a corresponding decrease in autophagy marker [22]. A similar phenomenon was observed in mitophagy. PINK1 and Parkin were used as the traditional mitophagy markers [23]. It was found that the expression of PINK1 and Parkin was increased in CSCs, and their involvement in mitophagy drives the expansion of tumor stem cell numbers [24, 25]. In addition to the traditional markers, the mitochondrial fission genes Drp1 and Fis1 can also regulate mitophagy affecting drug resistance in CSCs. The reduction in FIS1 impairs mitophagy and is detrimental to the survival of CSCs [26]. These observations suggest that autophagy and CSCs are closely related and that autophagy plays a critical role in regulating the multifarious functions of CSCs. However, to date, relevant mechanisms about the roles of autophagy in the CSCs have not been completely explained. The aim of this review was to summerize the potential impact of autophagy on the renewal, proliferation, differentiation, survival, metastasis, invasion and therapeutic resistance of CSCs and propose the related future perspectives.

## Role of autophagy in CSCs

### Autophagy can promote renewal and proliferation of CSCs

CSCs exhibit minimal differentiation and the ability to self-renew like the typical stem cells [27]. The different studies have demonstrated that autophagy at the basal levels is required to maintain the pluripotency of CSCs, and any deviation from the basal levels of autophagy can effectively reduce the renewal and proliferative properties of CSCs and promote senescence [19]. However, to fully comprehend the roles and uses of autophagy in CSCs, additional research is needed.

Investigations are currently ongoing to determine that how autophagy can potentially stimulate CSCs regeneration. For instance, prior studies have demonstrated that inhibition of autophagy can attenuate the renewal of CSCs in breast [28], pancreatic [29], and liver cancers [30]. Autophagy and nuclear factor erythroid 2–related factor 2 (NRF2) can effectively form a positive feedback regulatory loop to increase CSCs renewal by regulating ROS in ovarian cancer spheroid cells, and appropriate ROS levels contribute to the higher level of renewal efficiency [31]. Glucose transporter protein-1 (GLUT-1) and autophagy under hypoxic and low-glucose conditions can significantly enhance laryngeal CSCs proliferation [32]. Thus, reducing ATP synthesis to activate downstream A12MPK kinase and reversing the mTOR pathway can trigger autophagy and significantly inhibit the self-renewal ability of osteosarcoma stem cells (OSCs) [33]. It has been found that glutamine deficiency and inhibition of the autophagy consortium can lead to a substantial decrease in the number of CSCs in vivo [34]. TAp73 deficiency was reported to exacerbate glutamine dependence by decreasing superoxide dismutase 1 (SOD1) expression, thereby enhancing ROS accumulation, and increasing autophagy, thus significantly reducing glioblastoma self-renewal capacity [35]. MicroRNAs have also been shown to be involved in the regulation of autophagy. For example, miR-200b can inhibit RAB37 activity, suppress CSCs-mediated autophagy, and reduce the cell viability in glioblastoma CSCs [36]. In addition, studies have shown that the inhibition of ATG5 and ATG2B can suppress miR-181a, thereby reducing triple-negative breast cancer (TNBC) stemness [37]. MiR24-2 has also been found to enhance tyrosine kinase epigenetics in an autophagy-mediated manner, thereby promoting the malignant progression of hepatocellular carcinoma stem cells [38]. It has been also demonstrated that HULC through the autophagy-miR675-PKM2 pathway can upregulate expression of CyclinD1 to accelerate the growth of human hepatocellular carcinoma stem cells [39].

**Fig. 1:**
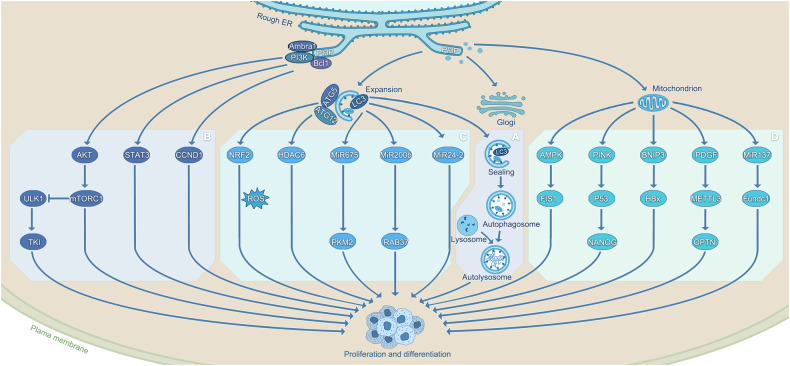
Autophagy promotes cancer stem cell renewal, proliferation and differentiation. A: The morphological process of autophagy mainly includes the formation of phagocytic vesicles, the formation of autophagosomes, and the formation of autolysosomes. B: Lupeol reduces CSCs differentiation through the PI3K/AKT/mTOR pathway [45]. AMBRA1 regulates the growth and proliferation of CSCs through the c-MYC/AMBRA1/STAT3 axis [41]. CCND1 can inhibit CSCs differentiation by inhibiting BCL1 [51]. Inhibition of ULK1 enhances TKI sensitivity and induces CSCs differentiation [50]. C: ATG5 and NRF2 form a positive feedback regulation loop, and CSCs update is increased by adjusting ROS [31]. HDAC6 mediated CSC growth inhibition is further enhanced under the induction of ATG12 [49]. HULC upregulates CyclinD1 through the LC3-miR675-PKM2 pathway to accelerate the growth of CSCs [39]. MiR-200b can inhibit RAB37 activity and LC3, reducing cell viability [36]. MiR24-2 also promotes the malignant progression of CSCs by enhancing the epigenetics of tyrosine kinase through LC3y [38]. D: AMPK-FIS1 pathway can affect the self-renewal of CSCs [26, 119]. When mitophagy is inhibited, p53 co-localizes with mitochondria. PINK1 binds to the NANOG promote to accelerate the growth of CSCs [135]. BNIP3L dependent mitophagy promotes enhanced CSCs activity induced by HBx [123]. PDGF-METTL3-OPTN can inhibit mitophagy to maintain their stemness [124]. MicroRNA-137 has also been demonstrated to maintain homeostasis by inhibiting mitophagy [121].

A number of different signaling pathways have been identified to play a key role in this process. It has been reported that TP73 deficiency can modulate the growth and stemness of CSCs through activation of the AMPK/TSC/MTOR signaling pathway [40]. The pro-autophagy factor AMBRA1 can regulate both the growth and proliferation of medulloblastoma stem cells through stimulating the c-MYC/AMBRA1/STAT3 axis [41]. Autophagy has also been shown to regulate the viability of gastric CSCs through targeting the Notch signaling pathway [42]. Autophagy can promote cancer cell proliferation and renewal through distinct signaling pathways and this has been documented in several prior studies. Interestingly, autophagy-dependent hepatocyte growth factor (HGF)-induced activation of Met/JNK and Met/STAT3 signaling in Axin2^+^ hepatocytes can increase CSCs renewal and proliferation [43]. It has also been reported that the inhibition of epidermal growth factor receptor (EGFR) signaling reduces SOX2 expression by promoting autophagic degradation, thereby enhancing the number of oral CSCs [44]. Moreover, several studies have demonstrated the negative effects of inhibiting autophagy on CSCs renewal and proliferation, but it was found that in retinoblastoma, lupeol promoted autophagy through the PI3K/AKT/mTOR pathway in retinoblastoma and reduced the number of retinoblast stem cells [45]. This observation contradicts the current mainstream view and reflects the "double-edged sword" effect of autophagy itself. This finding can be considered sufficient to give further thought to the fact that deviation from the basal levels of autophagy strongly reduces the renewal and proliferative properties of CSCs, whereas homeostasis of autophagy exerts a very important role in promoting the renewal and proliferation of tumor stem cells. Autophagy can differentially regulate the different breast CSCs through TGFβ/Smad and EGFR/Stat3 signaling, thereby limiting cancer growth [46]. With continuous improvements and breakthroughs in the detection tools, an increasing number of signaling pathways will surely be discovered, and thus the elimination of CSCs will surely be more efficient and precise (Fig. 1).

Interestingly, most of the relevant studies involved so far have focused on macroautophagy, and few have also explored the relationship between microautophagy or CMA and CSCs. CMA is a homeostatic process essential for the lysosomal degradation [12]. Recently, the potential relationship between CMA and glioma stem cells has been revealed. It was found that CMA activity directly depends on the level of LAMP2A, a key receptor for the CMA substrate protein at the lysosomal membrane, and targeted depletion of LAMP2A can effectively reduce glioblastoma stem cell (GSC)-mediated tumorigenic activity [47]. With the completion of this experiment, the mechanism of action between autophagy and CSCs has been thoroughly investigated.

### Autophagy provides cellular signals to promote the differentiation of CSCs

Although autophagy can alter this homeostasis by increasing CSCs differentiation, stem cells are generally long-lived and have efficient quality control mechanisms to balance the cell survival and tolerance to external and internal stimuli. A number of studies have revealed that the differentiation of embryonic CSCs results in the pronounced changes in protein homeostasis and cell survival, thus highlighting the importance of the PI3K-AKT-MTOR pathway, autophagic flux, and apoptosis regulation in maintaining their differentiation capacity [48]. HDAC6 suppression reduced CSCs pluripotency, whereas ATG7 and ATG12KD inhibition of CSCs multifunctionality can attenuate HDAC6 expression and stimulate differentiation. It’s interesting to note that the presence of inducers of autophagy can significantly improve the effect of HDAC6 KD on CSCs development [49]. It has also been demonstrated that the deletion of autophagy-inducing kinase ULK1 can substantially reduce the growth of xenografted chronic ML (CML) cells, ULK1-mediated inhibition of autophagy followed by enhanced TKI sensitivity was found to be driven by increased mitochondrial respiration and loss of quiescence, and oxidative stress induces leukemia stem cell differentiation and can contribute to the sensitivity of leukemia stem cells to targeted therapies [50]. Similarly, Cyclin D1 (CCND1) silencing reduced CD133, Becin-1, and LC3II expression in xenograft models of cancers, and inhibition of autophagy was found to suppress hepatocellular carcinoma stem cell differentiation [51].

BCSCs have been demonstrated to develop into endothelial cells under in vitro stimulation of vascular endothelial growth factor, and the blood vessels play a significant role in cancer growth and metastasis (VEGF). In BCSCs, Atg5 knockdown was observed to reduce the capacity for endothelial development [52]. High levels of CD44, ABCB1, and ADAM17 expression have been correlated significantly with poorer differentiation and greater malignancy grade in oral squamous cell carcinoma stem cells. This study demonstrated that autophagy controls the expression of CD44, ABCB1, and ADAM17 to suppress the emergence of cells with incomplete differentiation [53]. Autophagic flux was also found to be suppressed in TNB CSCs, and miRNA-181a expression was upregulated in TNB CSCs. Autophagy can inhibit the differentiation ability of TNB CSCs through miR-181a-mediated regulation of ATG5 and/or ATG2B [37]. These findings indicate that autophagy has an important role in regulating the differentiation of CSCs, and limiting the differentiation of the malignant cancer cells and improving the prognosis of cancer patients by inhibiting autophagy can have good clinical translational implications. The clinical translation of autophagy in CSCs is of great significance and needs to be further explored.

### Autophagy maintain the survival of CSCs

It’s still debatable how autophagy affects the development and spread of cancer. On the one hand, autophagy facilitates the survival of cancer cells to under unfavorable environmental conditions [54]; but on the other hand, it can lead to cell death once internal energy resources are depleted [11]. According to reports, FOXOs are essential for maintaining basal autophagy in the brain stem as well as progenitor cells and have been linked to autophagy [55]. Human pluripotent stem cells are more likely to survive when autophagy is stimulated by FOXO3A. However, whether autophagy also contributes to CSCs survival remains controversial [56]. Autophagy is crucial to the survival of CSCs, according to the related research. However, more research into the role of specific signaling pathways is required.

The role of autophagy in promoting CSCs survival is currently actively being investigated. Activation of mTOR expression can inhibit autophagy and promote apoptosis in head and neck squamous cell carcinoma [57] and as well as in stem cells [58]. Autophagy can stimulate GSK-3β/Wnt/β-linked protein signaling in colorectal cancer (CRC) to promote CSCs survival [59]. In acid-resistant glioma stem cells, by regulating the SDCBP/MDA-9/syntenin-mediated protective autophagy signaling pathway, it is feasible to shift the intracellular homeostasis from pro-survival to pro-cell death [60, 61]. Glutamate depletion-resistant prostate cancer cells employ autophagy as a defense mechanism against radiation-induced harm [62]. Similarly, a lack of glutamine leads to substantial inhibition of CSCs, whereas activation of ATG5 is able to resist radiation-mediated cell damage [34]. In OSSC, dysregulated accumulation of autophagosomes can trigger cell death [63]. A similar phenomenon occurs in pancreatic cancer, where endoplasmic reticulum-targeted alkyl phospholipid analogs can elicit a strong autophagic response in the pancreatic CSCs, for which the associated inhibition attenuates the protective autophagy [64]. In addition to inducing apoptosis and autophagic death in glioblastoma stem cells, the inhibition of autophagy-induced ferroptosis through lipid peroxide accumulation can markedly increase the sensitivity of CSCs to treatment [65].

However, just as autophagy plays a dual role in tumorigenesis, so does it play a dual one in CSCs survival. Autophagy causes CSCs death in contrast to the protective autophagy [66]. According to prior studies, MAKV-8 can substantially increase the acetylation of the target proteins, exerts cytotoxic and cytostatic effects, and concurrently initiates autophagy, which can result in cysteine-dependent apoptosis. Additionally, it has been demonstrated that the combination of MAKV-8 and imatinib can attenuate apoptosis by beclin-1 knockdown [67]. In another study, SAHA was shown to trigger autophagy through downregulation of AKT-mTOR signaling, thereby promoting apoptosis at an early stage [68]. In prostate cancer, autophagy could be induced through the activation of PI3K/Akt/mTOR signaling pathway, followed by apoptosis in the stem cells [69]. It was confirmed through studies that accumulation of autophagy leading to CSCs death was also observed in breast cancer [70], glioblastoma [71], and osteosarcoma [33]. Therefore, one cannot generalize the role of autophagy in the survival of CSCs; as autophagy itself is a regulatory mechanism of intracellular environmental homeostasis. Thus, it can potentially act as both protective autophagy, protecting CSCs from the lethal factors, and causing autophagic death or apoptosis to reduce the number of CSCs and increase susceptibility to the therapy. The complex role of autophagy increases the difficulty of treatment and simultaneously opens enormous avenues for development of novel therapeutic tools.

### Autophagy activates invasion and metastasis in CSCs

The correlation between epithelial–mesenchymal transition (EMT) and phenotypic differentiation of CSCs has been discussed in prior studies. For instance, it has been demonstrated that *H. pylori* infection induces chronic inflammation and EMT, and autophagy inhibitors can reduce the emergence of the mesenchymal phenotype and migratory capacity associated with EMT [72]. EMT in colorectal cancer is regulated by the SOX2-β-catenin/Beclin1/autophagy signaling axis in the colorectal CSCs [73]. These studies have demonstrated that autophagy and EMT are interrelated, and that cancer cells and CSCs undergoing EMT either have a high degree of overlap in the stimuli that induce their production or have similar functions, thus leading to a debate whether EMT cancer cells should be considered as CSCs [74].

With further research, it was found that autophagy is the key process for distinguishing CSCs from EMT cancer cells, and two models were proposed to describe the relationship between EMT cancer cells and CSCs [75]. The first was the branching model, in which the two possible outcomes exist: one is that paracrine factors in the cancer microenvironment can effectively promote the production of circulating CSCs by EMT cancer cells, and the other can result in the production of the non-circulating autophagic CSCs that adapt to changes in the cancer microenvironment by stimuli such as hypoxia. The second is a hierarchical model: EMT cancer cells can be induced to become autophagic CSCs in response to stimuli occurring in the cancer microenvironment, and once these stimuli are alleviated or replaced by the paracrine factors, autophagic CSCs can be transformed into circulating CSCs [76]. Both models predict the possibility of bidirectional transformation and phenotypic transformation also implies the functional transformation, that is, migration and the ability to metastasize [75].

Autophagy can also influence CSCs metastasis via a non-EMT approach. Knockdown of the pro-autophagy factor AMBRA1 in medulloblastoma, a powerful oncogenic signaling pathway, can reduce medulloblastoma stem cell growth and migration [41]. The autophagy-related factors DRAM1 and p62 have also been shown to regulate cell migration and invasion in glioblastoma stem cells [77]. Moreover, micro-changes in the tumor environment have an important influence on CSCs metastasis. In addition to the model-related effects discussed previously, autophagy inhibition under hypoxic and hypoglycemic conditions can also significantly reduce the proliferation and migration of CD133^-^positive laryngeal CSCs [32]. In contrast, induction of oxidative stress reduces autophagic activity in ovarian CSCs, can activate the onset of ferroptosis, and inhibit their proliferation, invasion, and tumorigenic capacity [78].

In conclusion, modulation of autophagy can significantly increase the ability of CSCs to invade, metastasize and become malignant. Although the crosstalk between EMT, autophagy, and CSCs has not been fully investigated, attenuation of tumor cell metastasis may be an excellent strategy for improving clinical patient prognosis, and hence it becomes necessary to continue to explore the mystery.

### Autophagy drives therapy resistance in CSCs

As described earlier, autophagy can affect the renewal, proliferation, differentiation, and metastasis of CSCs, thus increasing the survival of the cancer cells. However, CSCs can remain dormant for a long time and possess various drug-resistant molecules but are insensitive to external physicochemical factors that can kill cancer cells. Hence, cancers can often recur even after most common cancer cells are eliminated by conventional cancer treatments. Autophagy in CSCs can lead to the emergence of therapeutic resistance, which can be divided into (1) radiotherapy resistance, (2) chemotherapy resistance, and (3) immune resistance according to the current treatment.

Radiation therapy is known for its ability to activate the cytotoxic signaling pathways that ultimately can promote cancer cell death as well as numerous cytoprotective mechanisms triggered by the cellular injury [79]. Through signaling systems like CD98hc, autophagy was identified to be crucial in the development of cancer radioresistance [80]. Radioresistance in CSC has also been shown to be possibly associated with increased lysosome-mediated autophagy [81]. By inhibiting the process, it can increase the sensitivity of nasopharyngeal carcinoma stem cells (NPC) to the radiation therapy [82]. Radioresistant prostate CSCs have been shown to have a high glutamine requirement, and ATG5 activation in the absence of glutamine was found to resist radiation-mediated damage [34, 62]. Although research in this area is still scarce, autophagy may be considered a key process involved in the development of radiotherapy resistance in CSCs.

Drug chemoresistance is closely associated with the subpopulations of CSCs, and its activation is largely dependent on the activation of autophagy [83]. For instance, increased chemoresistance by autophagy has been demonstrated in renal cell carcinoma [84] and breast cancer [34]; however, the specific mechanisms have not been fully explored. GRP78 is a specific marker of chemoresistance in breast CSCs and thus blocking autophagy can reduce drug chemoresistance in breast CSCs through GRP78/β-linked protein/ABCG2 axis [85]. Similarly, the SOX2-β-catenin/Beclin1/autophagy signaling axis can promote chemoresistance in CRCSCs [73]. Moreover, other studies have found that autophagy in CRC cells can effectively promote chemoresistance in CRCSCs by activating GSK-3β/Wnt/β-linked protein signaling to enhance resistance to CRC therapy, whereas PIK3C3/VPS34 inhibitors can increase the efficacy of CRC therapy [59]. Inhibition of mTOR pathway can promote apoptosis in glioma stem cells and hepatocellular carcinoma [58, 86]. BRCA1 also regulates apoptosis and cell cycle progression through inducing autophagy, indirectly affecting drug sensitivity in ovarian CSCs [87]. Interestingly, autophagy can increase the sensitivity of glioblastoma stem cells to temozolomide not only by modulating apoptosis, but also by triggering ferroptosis through lipid peroxide accumulation [65]. In addition, there are evidences to suggest that autophagy plays a vital role in cytotoxicity. Autophagy could be induced by inhibition of Wnt pathway in breast cancer, which lead to increase in both the drug chemoresistance and survival of the tumor cells [88].

**Fig. 2:**
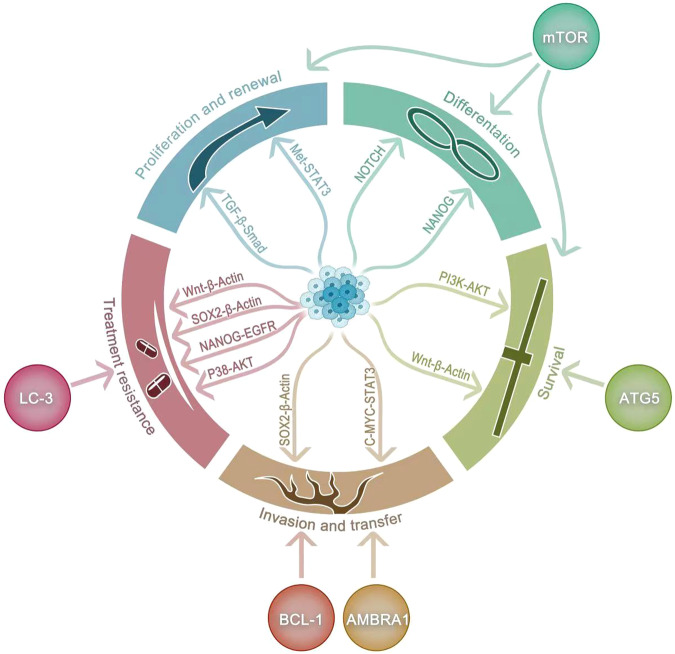
The role of autophagy in cancer stem cells. In summary, autophagy plays approximately five roles in cancer stem cells. Autophagy can affect NOTCH, NANOG, PI3K-AKT, SOX2 through LC3, BCL-1, Ambra1, ATG5, mTOR- β- Actin signaling pathways regulate the proliferation, renewal, differentiation, invasion, metastasis, survival, and drug resistance of cancer stem cells.

CSCs can also evade host immune surveillance. MiR20a-MICA/MICB signaling axis has been reported to evade NK cell-mediated killing [89]. In contrast, CSCs can downregulate ULBP ligands on NK cells during the dormancy and evade NK cell-mediated clearance [90]. CD47 molecule-mediated cancer cell self-protection is another potential mechanism of immune escape from CSCs, and inhibition of CD47 molecules can induce macrophage-mediated phagocytosis [91]. Interestingly, recent studies have shown that autophagic mechanisms can contribute significantly to immunosuppression-associated chemoresistance. It has been established that through the control of miR-155 and activation of TRAIL, autophagy inhibition increases CD4 cancer-infiltrating lymphocyte expression [92]. Therefore, it is possible that a deeper association exists between cancer cells, autophagy, and immunity. Autophagy can promote immune escape in pancreatic cancer through degradation of MHC-I [93], whereas autophagy under acidic culture can induce immunogenic cell death in bladder cancer cells, thereby promoting anticancer immunity, all of which clearly reflect the ability of autophagy to increase immune resistance of cancer cells [94]. Because CSCs are more relevant than the normal cancer cells and are often a major factor in cancer recurrence, studies have also explored how autophagy can mediate immune resistance in CSCs. For instance, previous studies have demonstrated that cytotoxic T lymphocyte (CTL)-mediated immune stress can transform NANOG CSCs to become immune-refractory and resistant to CTL [95]. LC3B upregulation in NANOG promotes immune resistance through stimulating over-activation of EGFR signaling, and the NANOG-LC3B-EGFR axis is a key factor in controlling NANOG immune-refractory cancers as a central molecular target [96]. It was also reported that HMBOX1 can inhibit the p38/AKT/mTOR pathway and its overexpression prevented liver cancer progression by promoting autophagy and increasing sensitivity to NK cell lysis [97]. Unlike common protective autophagy, autophagy plays a negative role here, which is consistent with the dual nature of autophagy, possibly due to the cytotoxicity caused by induced excessive autophagy. In bladder cancer, the ATG7/autophagy/FOXO3A/miR-145 axis has been identified as a novel molecular mechanism regulating PD-L1 mRNA stability [98]. Additional studies have shown that prostate CSCs can effectively improve androgen deprivation therapy (ADT) resistance by inhibiting the interaction of ATG7 and interleukin (IL)-6 receptors with the macrophages (TAM) [99]. Resistance to interferon-γ (IFN-γ)-induced autophagy may also be another important mechanism by which CSCs can resist immune eradication [100]. (Fig. 2) The crosstalk between autophagy, stem cells, and immune resistance still leaves many areas worth exploring, and is important for completely addressing the development of therapeutic resistance.

## Mitophagy’s "unique role" in CSCs: Preservation of stemness and plasticity

### Mitophagy facilitates the stemness of CSCs

As non-selective autophagy mechanisms have been completely explored, selective autophagy has gradually emerged as a hot spot for research in recent years [101]. Selective autophagy can be divided into different categories such as mitophagy [102], nuclear autophagy [103], endoplasmic reticulum autophagy [104] and lipophagy [105] depending on the target of its action. In contrast, mitochondria, as cellular energy hubs in eukaryotic cells, are susceptible to the damage caused by high levels of ROS thus leading to dysfunction and disruption of homeostasis [102]. Mitochondrial damage not only affects its own function, but may also adversely affect other organelles, proteins and membranes [106]. In turn, the Warburg effect exists in the tumor cells, and mitochondria, as the most important organelle in cellular metabolism, have important role compared to targeting other organelles [107, 108]. Therefore, among selective autophagy, mitophagy potentially may be of higher research value [109, 110].

Mitophagy, an important mechanism in the mitochondrial quality control system, can effectively degrade excess or damaged mitochondria through selective autophagy in response to the changes in the microenvironment [111]. Unlike conventional non-selective autophagy, mitophagy selectively targets mitochondria labeled by mitophagy receptors. Mitophagy, a crucial mechanism of mitochondrial quality control, generally performs a Inhibition role in advanced malignancies and an oncogenic role in the early stages of carcinogenesis [112, 113]. Although mitophagy’s significance in cancer has been the subject of numerous investigations, the mechanisms through which it affects CSCs have not yet been fully understood.

Additionally, related studies have found that PTEN-induced kinase 1 (PINK1) plays a key role in maintaining mitochondrial morphology and function by selectively degrading the damaged mitochondria. PINK1-dependent loss of mitophagy can significantly reduce the rate and efficiency of induced CSCs reprogramming [114]. When mitophagy is enhanced, p53 and mitochondria colocalize and are removed in a mitochondria-dependent manner. However, when mitophagy is inhibited, PINK1 can bind to the NANOG promoter to prevent the activation of NANOG expression by OCT4 and SOX2 transcription factors, thereby inhibiting the stemness and tumorigenic capacity of CSCs [115–117]. Similarly, inhibitors of mitophagy targeting p62 can significantly attenuate leukemia initiation potential in AML cells and impair the survival of leukemia-initiating cells (LIC) [118]. The mitochondrial kinetic regulator FIS1 mediates mitophagy in AML and lung cancer cells and depletion of FIS1 can cause attenuation of mitochondrial autophagy and lead to GSK3 inactivation, myeloid differentiation, cell cycle arrest, and severe loss of LSC self-renewal potential [26, 119]. The most potent autophagy inhibitor, Baf A16, can initiate mitophagy by reducing mitochondrial respiration and stabilizing PINK-1. The onset of stress, such as hypoxia, induces both mitochondrial damage and mitophagy, thus increasing the efficacy of autophagy inhibitors in inducing AML cell death [120]. It has been found that in the hypoxic microenvironment, microRNA-137 has also been shown to maintain homeostasis by inhibiting mitophagy in BCSCs-like cells (BCSLC) [121]. Mitophagy has also been found to contribute to increased drug resistance in CSCs, and silencing BNIP3L can significantly inhibit mitophagy by enhancing the degree of sensitivity of colorectal CSCs to DXR [122]; whereas BNIP3L-dependent mitophagy promoted HBx-induced enhancement of hepatocellular carcinoma stem cell activity [123]. It was also found that PDGF-METTL3-OPTN inhibited mitophagy in glioblastoma stem cells to maintain the stemness [124]. In conclusion, in CSCs, mitophagy can maintain stemness by degrading abnormal mitochondria, decreasing intracellular ROS levels, scavenging oncogenes, and exerting other effects on stemness, drug resistance, and increasing adaptability to microenvironmental alterations of CSCs to different degrees, which play a very important role in tumorigenesis.

### Mitophagy facilitates the maintenance of plasticity

TME promotes plasticity and ultimately confers resistance to different chemotherapeutic agents in CSCs [125]. The role of autophagy in the ecological niche of CSCs provides metabolic plasticity in hypoxic, energy-deprived CSCs [126]. The control of mitochondrial activity, which has tremendous impact on the stemness, longevity, and metastatic potential of CSCs, is essential for ATP synthesis and metabolic reprogramming [11, 127]. The cancer cells are predominantly dependent on aerobic glycolysis, a phenomenon known as the "Warburg effect" [128]. Although some studies have described that CSCs can also be driven by glycolytic reprogramming [129], there is growing evidence to suggest that they rely primarily on oxidative phosphorylation (OXPHOS) for energy [130, 131]. It has been shown that CSCs increases the antioxidant defense against ROS production by increasing the rate of OXPHOS [131]. Interestingly, by destroying the damaged mitochondria that are linked to it, mitophagy can control the ROS levels and prevent the induction of programmed cell death [132]. OXPHOS is the primary source of energy for human pancreatic CSCs, and higher mitochondrial activity raises CSCs stemness [133]. The stemness of hepatic CSCs can be maintained by removing p53 localized to the mitochondria in a mitochondria-dependent manner [134].

In addition, mitophagy can regulate hepatic CSCs by promoting the transcriptional activation of NANOG [135]. CSCs can trigger a shift from OXPHOS to glycolytic metabolism, thereby increasing their activity and self-renewal potential [136]. Under hypoxic conditions, CSCs can utilize BNIP3- or FUNDC1-dependent activation of HIF-1α mitophagy, which can mediate the metabolic shift to glycolysis [137, 138]. Dichloroacetate is a known anticancer agent that can cellular metabolism from anaerobic glycolysis to OXPHOS by inhibiting pyruvate dehydrogenase kinase, and it has also been shown that mitophagy can be involved in counteracting the toxicity of CSCs [139].

However, excessive mitophagy has also been shown to increase drug resistance in CSCs. For example, in colorectal cancer cells, CSCs were shown to resist adriamycin-induced cell death through BNIP3L-mediated mitophagy [122]. In conclusion, the metabolic plasticity of the CSCs is essential for its survival. Metabolic remodeling through mitophagy and conversion to glycolytic or OXPHOS phenotypes can facilitate metabolic reprogramming, which, in turn, can promote cancer survival and progression. Mitophagy, a mitochondrial quality control modality, is likely to be involved in the compensation of CSCs for the metabolic changes and its protection to adapt to metabolic shifts. Overall, the mechanisms of the interaction between mitophagy and CSCs metabolism needs to be studied in more detail.

## Clinical application of autophagy in CSCs

### The use of autophagy inducers in CSCs

As per the double-edged role of autophagy already discussed above, on one hand, it can function to prevent the occurrence of these damages and eventually suppress tumorigenesis by eliminating the damaged organelles and chromosomes. In contrast, autophagy is a defense mechanism used by cells to survive in a hostile environment. Rapidly proliferating cancer cells require more energy, and activation of autophagy can provide them sufficient energy to promote their aberrant growth. Therefore, a combination of drugs targeting CSCs and autophagy inhibitors is expected to improve the effectiveness of cancer treatment. Gastric CSCs treated with autophagy inhibitors were found to reduce the mesenchymal phenotype associated with EMT, the emergence of migratory capacity, CD44 expression, and the ability to form cancer spheres [72]. By modifying the cancer microenvironment, autophagy inhibitors have also been demonstrated to drastically lower CSCs viability in gastric cancer [140]. The development and metastasis of cancer can be significantly influenced by the blood arteries. Some CSCs could develop into endothelial cells and support angiogenesis. BCSLC, an autophagy inhibitor, can also reduce angiogenesis by preventing endothelial differentiation [52].

It’s interesting to note that head and neck squamous cell carcinomas were rendered more susceptible to the apoptotic effects of afatinib when it was used in combination with an autophagy inhibitor and a tyrosine kinase inhibitor [57]. Chloroquine (CQ) or hydroxychloroquine (HCQ) treatment is the most commonly used autophagy inhibitor clinically [141]. The use of CQ could reduce the activity, number, tumorigenicity, and resistance to gemcitabine in CSCs obtained under unfavorable conditions [22]. CQ significantly increased the apoptosis of CD133+ hepatocellular carcinoma stem cells, thus rendering the liver CSCs more sensitive to alterations in the cancer microenvironment, such as hypoxia and nutrient deficiency, thereby contributing to improved anticancer therapy [142]. CQ could also induce mitochondrial damage, leading to mitochondrial membrane depolarization, causing a significant decrease in cytochrome c oxidase activity, and accumulation of superoxide and double-stranded DNA breaks, which effectively reduced the ability of TNBC cells to metastasize. Moreover, when co-administered with carboplatin, it effectively inhibited carboplatin-induced autophagy, significantly reduced the expression of CSCs subpopulation DNA repair proteins in TNBC, and resulted in cancer growth reduction in carboplatin-resistant TNBC [143].

It has been also demonstrated that when LGR5^(+)^ colorectal CSCs were co-treated with curcumin and an autophagy inhibitor (HCQ), curcumin-induced inhibition of cell proliferation in LGR5^(+)^ colorectal CSCs was significantly reduced [144]. Lys05, dimeric quinapine (DQ661), conamycin A, protease inhibitor E64d, V-ATPase inhibitor of gastrin A, 3-methyladenine, and GNS561 are classes of new generation lysosomal inhibitors. The application of these novel autophagy modulators adds more possibilities for the treatment of CSCs [145]. Although the autophagy activator rapamycin had the opposite effect, silencing LETM1 caused autophagy in CRC cells by inducing ROS-mediated AMPK/mTOR signaling pathway, thereby preventing the progression of CRC. The autophagy inhibitor 3-methyladenine reversed the inhibitory effect of LETM1 silencing on proliferation and renewal of colorectal CSCs through ROS-AMPK-mTOR axis [146]. GNS561 can significantly reduce the number of CSCs by specifically inhibiting palmitoyl protein thioesterase 1 (PPT1), thus impairing histone protease activity and reducing autophagic flux [30, 147]. It is encouraging to note that GNS561 has just been approved for global phase 1b clinical trial in liver cancer and might have a high clinical translation value in the future. Lys05-mediated autophagy suppression in chronic myelogenous leukemia (CML) can decrease LSC quiescence and promote myeloid cell proliferation. In addition, Lys05 can reduce the numbers of LSC in CML target xenografts when used in conjunction with TKI therapy. These findings offer a compelling case for selecting potent second-generation autophagy inhibitors as efficient CSCs targets [54] (Table 1).

**Table 1:** Clinical application of autophagy in cancer stem cells.

Category	Type of Cancer	Drugs used	Role	Reference
Autophagy inducers	Gastric Cancer	Bafilomycin, Chloroquine	Reduces EMT-related phenotype, migration capacity, CD44 expression, and tumorigenic capacity	[71]
	Gastric Cancer	Chloroquine	Reduced cancer stem cell viability under altered tumor microenvironment	[139]
	Breast Cancer	Chloroquine	Decreased endothelial differentiation and reduced angiogenesis	[51]
	Squamous cell carcinoma of the head and neck	Afatinib	Combination of tyrosine kinase inhibitors increases sensitivity of cancer cells to them	[56]
	Squamous cell carcinoma of the head and neck	Hydroxychloroquine	Reduced resistance to gemcitabine	[141]
	Liver Cancer	Chloroquine	Resulting in greater sensitivity to alterations in the tumor microenvironment	[142]
	Liver Cancer	GNS561	Reduced tumor growth and decreased the frequency of CSCs	[29]
	Colorectal Cancer	Kaplan	Reduced cell proliferation of CSCs	[143]
	Colorectal Cancer	Curcumin and hydroxychloroquine	Reduces stem cell proliferation and renewal	[144]
	Colorectal Cancer	3-Methyladenine	Inhibition of AMPK / mTOR pathway blocks CRC progression	[145]
	Colorectal Cancer	Rapamycin	Lys05 in combination with tyrosine kinase inhibitors reduces the number of CML stem cells	[145]
Immunotherapy	Bladder Cancer	Actinomycin D	PD-L1 and autophagy inhibitor combination therapy reduces tumor stem cell population	[94]
	Liver Cancer		HMBOX1 promotes autophagy, suppresses CSC phenotype, and increases sensitivity to NK cell lysis to inhibit hepatocellular carcinoma growth	[96]
	Liver Cancer		Hepatocellular carcinoma CSC increases immune resistance through resistance to IFN-γ-induced autophagy	[99]
	Liver Cancer	Chloroquine	Novel lysing adenovirus of Wnt signaling acts on apoptosis and autophagy to effectively inhibit cancer stem cell growth	[150]
	Stomach Cancer	Chloroquine	IL-17B/IL-17RB signaling cascade regulates Beclin-1 ubiquitination and promotes cancer stem cell self-renewal and tumorigenesis	[148]
	Prostate Cancer		Cancer stem cell-like cells and macrophages act together to promote prostate cancer progression, while CSCs improve ADT resistance by inhibiting the interaction of ATG7IL6 receptors with TAMs	[98]
Metabolic Therapy	Prostate Cancer	Chloroquine	Glutamine deprivation leads to a decrease in the number of CSCs, while modulation of redox status and ATG5-mediated autophagy can be used as a survival strategy against radiation-induced damage	[33, 61]
	Breast Cancer	Chloroquine	Inhibition of autophagy induces mitochondrial structural damage and double-stranded DNA break repair damage to reduce the growth of cscs	[143]
	Breast Cancer	Salinomycin	Induces ROS-mediated apoptosis, inhibits lysosomal activity and autophagic flux, and reduces the tumorigenic capacity of cscs	[155]
	Breast Cancer		Affecting lipid metabolism and thus inhibiting autophagy affects its stemness	[152]
	Glioblastoma	Oligomycin A, antimycin A	Mitochondrial inhibitors promote the death of glioma stem cells by inhibiting autophagy	[153]
	Esophageal Cancer	Chloroquine	Induction of apoptosis in esophageal cancer stem cells by alcohol dehydrogenase, while inhibition of autophagy increases its effect	[154]

Currently, the most widely used strategy in this field is the use of autophagy modulators to directly affect autophagy and thus the properties of CSCs. However, compared to the usual autophagy inhibitors, lysosomal inhibitors appear to be more novel. However, it is worth considering that lysosomal inhibitors act mainly to affect the degradation of autophagic vesicles and exert little effect on the isolation of cargoes such as mitochondria. Therefore, lysosomal inhibitors might be less effective in treating tumor cells that rely on mitochondrial autophagy. Moreover, there are no studies describing the concentration of autophagy modulators that should be used, which may depend on the different types of tumors and their dependence on autophagy. In the future, with the birth of more novel autophagy modulators, it is expected that there is a high probability to improve the therapeutic efficacy against malignant tumors.

### Crosstalk role between autophagy and immunotherapy in CSCs

It has been found that in-depth research on the potential relationship between autophagy and immunity in CSCs have established that immune selection causes cancer cells to develop resistant phenotypes. Although reduction of LC3B in immune-refractory cancer models can render malignancies susceptible to pericyte metastasis and PD-1/PD-L1 inhibition, resulting in successful long-term control of tumors like colorectal cancer. Cytotoxic T lymphocyte (CTL)-mediated immunological stress can render NANOG CSCs resistant to CTL [95, 96]. Thus, application of combination therapy with PD-1/PD-L1 and autophagy inhibitors can enhanced human basal cell carcinoma treatment compared with immune checkpoint inhibitors alone [98]. The expression of a novel transcriptional repressor, Homeobox (HMBOX1), in hepatocytes can potentially increase the sensitivity of cancer cells to NK cell lysis by promoting autophagy, thereby suppressing the CSCs phenotype and inhibiting hepatocellular carcinoma cell progression [97]. Thus, by controlling Beclin-1 ubiquitination, the IL-17B/IL-17RB signaling cascade can promote CSCs self-renewal and cancer [148]. Additionally, it has been shown that CD133^+^ HCC CSCs can resist IFN-induced autophagy, suggesting that this may also be a way for CSCs to fend off immune elimination [100]. The crosstalk between CSCs and macrophages can promote prostate cancer progression and ADT resistance compared to targeting CSCs alone, thereby providing a plausible approach to improve ADT resistance in prostate cancer [99]. Lysovirus (oncolytic virus [OVs]) therapy is a new type of anticancer therapy [145], which can interfere with the essential autophagic systems, and survive as well as spread within cancer cells [149]. It was found that a new lysing adenovirus that targets Wnt signaling can effectively stop the formation of CSCs-like cells by affecting apoptosis and autophagy [150]. Additionally, by activating the danger signaling molecules, the adenovirus E4 protein can suppress autophagy, induce autophagy-associated immunogenic cell death, and strengthen the immune system’s ability to fight cancer [151].

In summary, autophagy is able to enhance the killing ability of tumor cells through combined immunotherapy. However, detailed mechanisms about the role of autophagy among immune cells and tumor stem cells still remain to be explored. Immunotherapy is currently the hottest area in tumor treatment, and empowering overt immune cells such as CAR-T or CAR-M to induce autophagy can be developed as an important strategy for clinical treatment of tumors in the future.

### Autophagic therapy for targeted metabolism in CSCs

The pathways regulating CSCs metabolism include those related to glutamine, mitochondrial, and lipid metabolism [152]. It is important to explore additional therapeutic tools by investigating the mechanisms of the interaction between autophagy and metabolism in CSCs. It has been reported that prostate cancer cells can increase the efficacy of radiation therapy through glutamine deprivation, which can lead to DNA damage and depletion of CSCs [62], and autophagy mediated by regulation of redox status as well as ATG5 can be used as a survival strategy against radiation-induced damage. Thus, the combination of targeted glutamine metabolism and autophagic radiosensitization can be an effective tool for the treatment of CSCs [34]. The autophagy inhibitor CQ can effectively target CSCs by inhibiting autophagy and inducing mitochondrial structural damage and double-stranded DNA break repair damage [143]. Interestingly, similar studies have also found that mitochondrial inhibitors, such as oligomycin A and antimycin A, can increase the cytotoxicity of GSCs by inhibiting autophagy, thereby providing a potential therapeutic option for targeting mitochondria in glioblastoma [153]. Moreover, induction of mitochondrial superoxide production and oxidative stress associated with mitochondrial depolarization via alcohol dehydrogenase metabolism can induce substantial apoptosis in esophageal CSCs, whereas inhibition of autophagy can increase ethanol-mediated apoptosis, which also reflects the protective effect of autophagy [154]. It has been also demonstrated that salinomycin (Sal) induced ROS and mitochondrial pathway-mediated cell apoptosis suppressed lysosomal activity, as well as autophagic flux, and reduced the carcinogenic capacity of BCSCs [155]. In breast cancer, in addition to inhibiting mitochondrial metabolism, it can also affect lipid metabolism, and thus inhibit autophagy to affect its stemness [152]. In conclusion, glutamine, mitochondrial, and lipid metabolism can aid autophagy to modulate the therapeutic effect of cancers; however, in general, the excavation of related fields is still not deep enough, and further studies are needed to discover their possible interrelationships.

## Conclusion and future perspectives

CSCs play a very important role in regulating cancer proliferation, renewal, metastasis, and chemotherapy resistance. They can remain dormant for a long time and insensitive to various external microenvironmental changes, leading to the development of drug resistance. Therefore, CSCs are considered a key point to overcome important bottlenecks in the field of cancer therapy. However, targeting and eliminating malignant CSCs without affecting the normal stem cells of the organism is a difficult task and can hamper the development of novel cancer therapy. It has been shown above that autophagy plays an important role in the maintenance, metastasis, as well as therapeutic resistance of CSCs, and it has different roles in CSCs at different stages of cancer. During the early stages of cancer, the main function of autophagy is to inhibit CSCs formation. But, in the middle and late stages of cancer, autophagy plays a protective role against changes in the cancer microenvironment, inhibits the apoptosis of CSCs, and promotes the survival of the cancer cells. However, whether autophagy plays different roles in different cancers or different autophagy genes can play distinct roles remain to be explored.

As a hot research topic in recent years, many researchers have explored the link between mitophagy and CSCs, but no key breakthrough has yet been achieved. What are the constraints associated with mitophagy and CSCs experiments compared with previous research hotspots of macroautophagy? Recent experiments on CMA and CSCs have made relevant progress, but does this open new perspectives on the potential link between autophagy and stem cells? Should future research hotspots in this field continue to focus on macroautophagy or shift to other types of autophagy? Lipid metabolism has also been a hot topic of research in recent years, and thus another relevant question is whether there is a close connection between lipophagy and CSCs? How do autophagy and apoptosis interact together in CSCs? All these questions need to be answered through further research.

With improvements in science and technology, both immunopharmaceutical therapy and nanomaterials are being increasingly used in cancer therapy in combination with autophagy modulators. Novel assays of autophagic flux can also be used for exploring the relationship between the two. However, there are still some technical limitations to these studies. It is worth noting that neither the optimal dose of autophagy inhibitors nor the possible functional differences resulting from the phenotypic changes in autophagy genes have been consistently determined. More importantly, most of the existing studies have been conducted on preclinical models, and more clinical studies are needed to corroborate the results of these experiments. It is important to optimize and improve cancer treatment protocols by exploring the mechanism of interaction between autophagy and CSCs.

## Supplementary information

aj-checklist

## Data Availability

All data in this article comes from public databases such as PubMed.
